# Understanding the Spatial Topology of Artificial Immunological Synapses Assembled in T Cell-Redirecting Strategies: A Major Issue in Cancer Immunotherapy

**DOI:** 10.3389/fcell.2019.00370

**Published:** 2020-01-10

**Authors:** Pedro Roda-Navarro, Luis Álvarez-Vallina

**Affiliations:** ^1^Department of Immunology, Ophthalmology and ENT, School of Medicine, Universidad Complutense, Madrid, Spain; ^2^Lymphocyte Immunobiology Group, Instituto de Investigación Sanitaria 12 de Octubre (imas12), Madrid, Spain; ^3^Cancer Immunotherapy Unit (UNICA), Department of Immunology, Hospital Universitario 12 de Octubre, Madrid, Spain; ^4^Immuno-Oncology and Immunotherapy Group, Instituto de Investigación Sanitaria 12 de Octubre (imas12), Madrid, Spain

**Keywords:** immunological synapse, cancer, immunotherapy, CAR T cell, bispecific antibodies

## Introduction

T cell-redirection strategies aim to selectively eliminate cancer cells by physically linking T lymphocytes with cancer cells using tumor-targeted *cell-cell bridging* (CCB) molecules, such as membrane-anchored *chimeric antigen receptors* (CARs) or soluble *bispecific antibodies* (bsAbs) that specifically recognize a cell-surface *tumor-associated antigen* (TAA) (Blanco et al., 2019). In the CAR approach, a TAA-specific antibody is genetically fused to intracellular T cell signaling domains. CARs have evolved greatly since their initial description, as single-chain antibody fragment (scFv)-based receptors containing the signaling domain of the CD3ζ chain (CD247) of the *T cell receptor* (TCR) (Eshhar et al., 1993). Subsequently, constructs incorporating signaling domains of costimulatory molecules (e.g., CD28 or 4-1BB) in tandem with the CD3ζ signaling domain were generated (Finney et al., 1998). Engrafting T-cells with such receptors, termed second-generation CARs, enables sustained proliferation and increased cytokine secretion. Third-generation CARs contain two costimulatory domains, in addition to the CD3ζ signaling domain (Carpenito et al., 2009; Milone et al., 2009). Current CAR-T cell therapy involves the isolation of autologous T cells using leukapheresis, followed by *in vitro* stimulation, genetic modification to express the TAA-specific CAR, and expansion to infuse back into the patient (Blanco et al., 2019). The bsAbs are designed to simultaneously bind to the TAA in the surface of tumor cells and the CD3ε chain of the TCR/CD3 complex in the surface of T cells (Blanco et al., 2019). More than a 100 different bsAb formats have been reported, including small bsAbs composed only by two antigen-binding sites, IgG-like bsAbs and larger and non-IgG bsAbs formed by different antigen-binding moieties, often combined with oligomerization modules (Nuñez-Prado et al., 2015; Brinkmann and Kontermann, 2017). By connecting CD3 signaling molecules with a recognition process independent of the TCR variable domains, T cells can be hot-wired to recognize a user-defined cell-surface TAA that is not associated with the *major histocompatibility complex* (MHC) to activate effector cell responses and kill cancer cells (Blanco et al., 2019). Nonetheless, the precise molecular mechanisms by which T cells are activated through these CCB molecules are poorly understood.

The administration of bsAbs and CAR-T cells has achieved remarkable clinical outcomes in hematological tumors, and several products have been approved by regulatory agencies for clinical use. Blinatumomab, an anti-CD19xanti-CD3 bsAb designed in the BiTE (*bispecific T cell-engager*) format, was approved by the US Food and Drug Administration (FDA) for the treatment of relapsed or refractory B cell acute lymphoblastic leukemia (B-ALL) (Przepiorka et al., 2015). Two CD19-specific second generation CAR-T cell products, tisagenlecleucel and axicabtagene ciloleucel (axi-cel), have been approved by the US FDA for the treatment of pediatric and young adult patients with relapsed or refractory B-ALL (Maude et al., 2018) and adult patients with relapsed or refractory diffuse large B cell lymphomas (Neelapu et al., 2017), respectively. However, the utility of these approaches in the treatment of solid tumors targeting TAAs has been limited by organ toxicities related to activation of T cell effector functions by non-tumor cells expressing low levels of the TAA, as well as systemic cytokine-associated toxicities (Alonso-Camino et al., 2016).

## Physiological T Cell Activation and the Immunological Synapse

**Figure 1:**
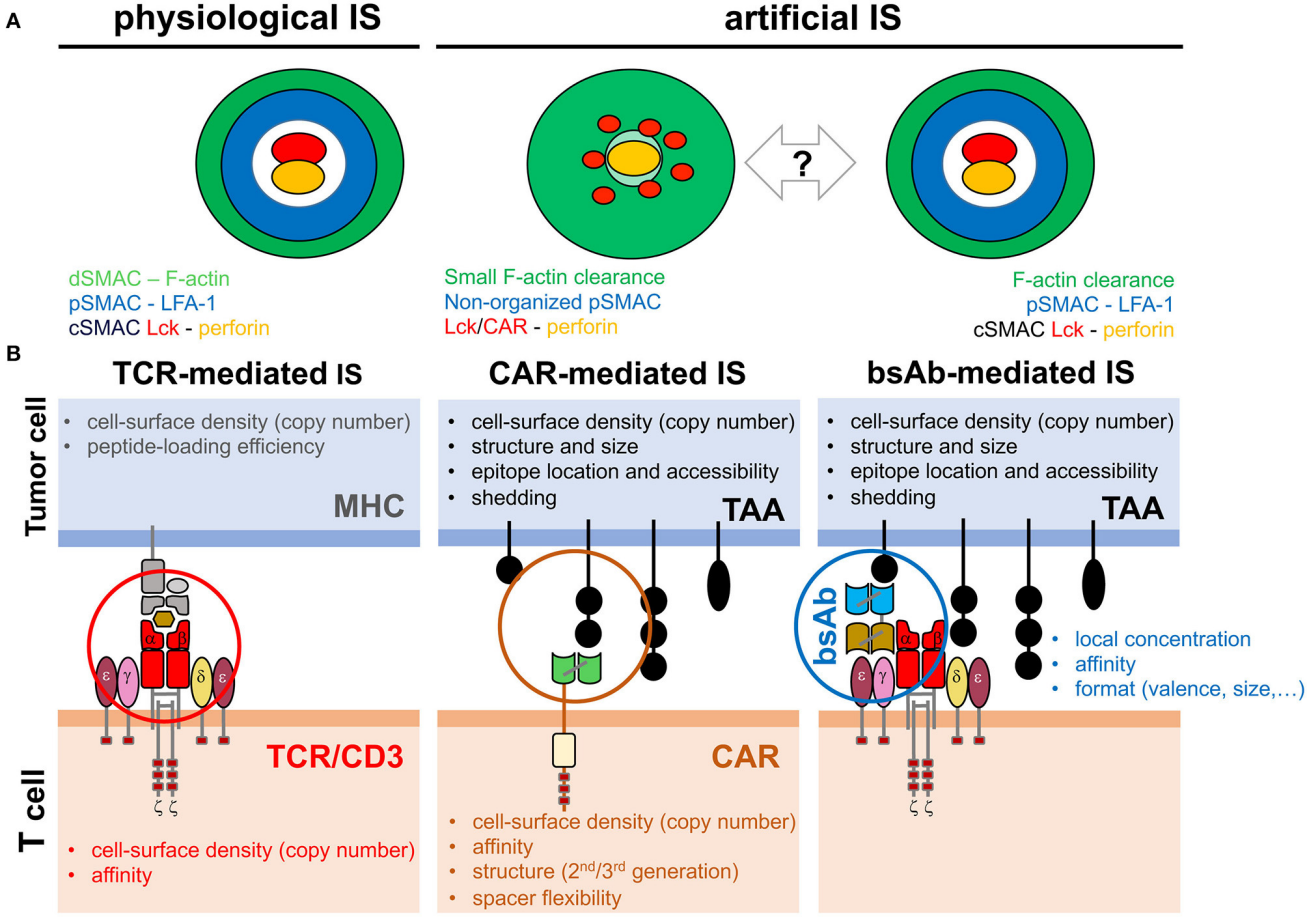
Factors potentially affecting IS topology. **(A)** Reported topology observed in the TCR-mediated, CAR-mediated and bsAb-mediated IS. The organization of cytoskeleton, signaling and effector molecules along the interface of the IS established by T cells and target cells is depicted. The TCR-mediated and the bsAb-mediated IS display a well-organized bull's eye structure whereas CAR-mediated IS shows a rather disordered structure. Bidirectional arrow and question mark indicate the uncertainty of expected topology in future CAR and bsAb formats. **(B)** Schematic of the activating system in TCR-mediated physiological IS or CCB-mediated artificial IS. The different factors influencing IS assembly and T cell activation are indicated in the tumor cell and in the T cell.

Under physiological conditions, TCR engagement leads to suppression of T cell locomotion and formation of the *immunological synap*se (IS), a highly organized structure at the interface between antigen-presenting cells or target cells and T cells (Alcover et al., 2016). The TCR-mediated IS (TCR-IS) is currently seen as a three-dimensional dynamic structure in which the endosomal compartment, the cytoskeleton, and the signaling network are finely tuned to achieve proper T cell activation and effective immune responses (Soares et al., 2013). The mature TCR-IS is compose of a central *supramolecular activation cluster* (cSMAC), a peripheral (p)SMAC and a distal (d)SMAC (Figure 1A) (Monks et al., 1998; Freiberg et al., 2002). The cSMAC is the docking site for the *microtubule organizing center* (MTOC), which generates a radial net of microtubule fibbers that cover all the IS and mediates the polarization of the endosomal compartment (Martín-Cófreces et al., 2014). pSMAC and dSMAC are sites where contractile actomyosin arcs and an actin retrograde flow, respectively, generate centripetal mechanical forces toward the cSMAC (Babich et al., 2012; Murugesan et al., 2016; Basu and Huse, 2017). In this context, early signaling of the TCR/CD3 complex in response to a strong agonist occurs in plasma membrane microclusters at the dSMAC that move to the cSMAC where the TCR is endocytosed and early signaling ceases (Varma et al., 2006). However, in the case of weak agonist, cSMAC has been proposed to enhance T cell activation (Cemerski et al., 2008). The centripetal movement of TCR microclusters toward the cSMAC is mediated by the actin retrograde flow at the dSMAC, by the contractile actomyosin arcs at the pSMAC and by dynein motors on microtubule fibbers (Hashimoto-Tane et al., 2011; Yi et al., 2012; Murugesan et al., 2016). Cytoskeleton dynamics are also essential for an adequate T cell activation. For instances, the actin retrograde flow at the dSMAC sustains the PLCγ1 activation (Babich et al., 2012) and MTOC polarization to the IS controls sustained activating signals (Martín-Cófreces et al., 2008).

Sustained TCR/CD3 signaling is required to achieve a proper T cell activation and the endocytosed and degraded TCR during activation should be replenished by TCR molecules recruited to the IS from the endosomal compartment (Das et al., 2004). The endosomal compartment also conveys signaling molecules, such as Lck and LAT, which participate in signaling complexes organized at the IS (Ehrlich et al., 2002; Bonello et al., 2004; Purbhoo et al., 2010; Balagopalan et al., 2018).

During T cell effector functions, the cSMAC is the place from where lytic granules or cytokines are secreted to the synaptic cleft by cytotoxic or helper T cells, respectively (Huse et al., 2006; Stinchcombe et al., 2006). This role depends on the actin clearance and the polarization of the MTOC to the cSMAC, as well as on integrin rings at the pSMAC that ensure the required cell-cell adhesion (Martín-Cófreces et al., 2018).

## Immunological Synapse Mediated by CCB Molecules

The precise spatial and temporal topology of the IS assembled in response to CCB molecules (CCB-IS) is poorly understood. Although CAR-T cell stimulation induces an efficient MTOC polarization and lytic granule secretion (even faster than in TCR-IS), actin cytoskeleton is not completely depleted from the center of the IS, microclusters of the CAR and signaling molecules are evenly dispersed through the IS, cSMAC and LFA-1 ring at the pSMAC are not properly organized and activating signals as well as cell-cell interactions are shorter than in conventional TCR-IS (Figure 1A) (Mukherjee et al., 2017; Davenport et al., 2018; Watanabe et al., 2018). It should be noted that this fast CAR-T-IS might be instrumental in the secretion of lytic granules before MTOC polarization to the IS, as previously observed (Bertrand et al., 2013). In the case of bsAb-mediated IS, initial work indicates the establishment of a conventional mature IS with a typical ring of LFA-1 at the pSMAC and polarization of the secretory compartment at the cSMAC (Figure 1A) (Offner et al., 2006). Interestingly, filamentous actin, CD3ζ-containing endosomes and PLCγ1 activating signals properly polarize to the bsAb-mediated IS (Harwood et al., 2017).

## Discussion

In spite of the above-mentioned recent contributions, a precise understanding of the spatial and temporal topology of the CCB-IS remains elusive. To what degree is the physiological IS organization maintained in the artificial CCB-IS formed in CAR-T cells or following CD3ε engagement by a TAA-bound bsAb? This question is particularly relevant as an altered topology of the TCR-IS has been associated with immune pathologies (Schubert et al., 2012). Structural or procedural differences between the CCB-IS and TCR-IS will likely change the nature of the resulting signaling and influence therapeutic T cell responses. Thus, in cancer immunotherapy, the study of the CCB-IS is expected to improve the efficiency of the treatment while reducing side-effects. Consistent with this idea, the IS seems to predict the efficiency of CAR-T cells (Xiong et al., 2018). Considering the cancer cell side, conventional T cell activation and TCR-IS assembly is mainly influenced by the copy number of the MHC (frequently reduced by cancer cells) and by the affinity of the MHC/peptide engagement by the TCR. In the artificial CCB-IS many other factors can be influential, such as the TAA density (copy number and shedding), size and structure, the location of the targeted epitope (accessibility and distance to the cell membrane), and the number and affinity of crosslinking events mediated by CCB molecules. Other decisive factors are the structure and format (size, geometry and valence) and the density or local concentration of the CCB molecule (CAR or bsAb) (Figure 1B).

The influence of some of these factors in the assembly of the CCB-IS has been studied. For example, in the bsAb-mediated IS membrane proximal TAA-epitopes are necessary for the assembly of the IS with CD45 exclusion and central clustering of the TAA, the signaling molecule ZAP70 and the bsAb (Li et al., 2017). Also, an incremented TAA binding valence of bsAbs contributes to a more efficient activating signaling at the IS and effector function (Harwood et al., 2017). Nonetheless the precise spatial and temporal topology of intracellular signaling and cellular organelles following lymphocyte activation with different formats of CCB molecules should be deeply studied. Such information will allow us to know which is the strategy that best reproduces the molecular mechanisms underlying canonical TCR-mediated activation and effector function, as well as to determine whether it is possible to improve the tumoricidal potency of T cells redirected to the tumor by CCB molecules, with limited collateral damage to normal tissues. Thus, studies to understand IS topology must be included in the roadmap for the development of safer and more effective T cell-redirecting strategies for cancer immunotherapy.

## Author Contributions

PR-N and LÁ-V contributed to the conception of the work and wrote the manuscript.

### Conflict of Interest

The authors declare that the research was conducted in the absence of any commercial or financial relationships that could be construed as a potential conflict of interest.

## References

[B1] Alcover A. Di Bartolo V. Roda-Navarro P. (2016). Editorial: molecular dynamics at the immunological synapse. Front. Immunol. 7:632. 10.3389/fimmu.2016.00632 28066441 PMC5174117

[B2] Alonso-Camino V. Harwood S. L. Álvarez-Méndez A. Alvarez-Vallina L. (2016). Efficacy and toxicity management of CAR-T-cell immunotherapy: a matter of responsiveness control or tumour-specificity? Biochem. Soc. Trans. 44, 406–411. 10.1042/BST20150286 27068947

[B3] Babich A. Li S. O'Connor R. S. Milone M. C. Freedman B. D. Burkhardt J. K. (2012). F-actin polymerization and retrograde flow drive sustained PLCγ1 signaling during T cell activation. J. Cell Biol. 197, 775–787. 10.1083/jcb.201201018 22665519 PMC3373411

[B4] Balagopalan L. Yi J. Nguyen T. McIntire K. M. Harned A. S. Narayan K. (2018). ma membrane LAT activation precedes vesicular recruitment defining two phases of early T-cell activation. Nat. Commun. 9:2013 10.1038/s41467-018-04419-x 29789604 PMC5964120

[B5] Basu R. Huse M. (2017). Mechanical communication at the immunological synapse. Trends Cell Biol. 27, 241–254. 10.1016/j.tcb.2016.10.005 27986534 PMC5367987

[B6] Bertrand F. Müller S. Roh K. H. Laurent C. Dupré L. Valitutti S.. (2013). An initial and rapid step of lytic granule secretion precedes microtubule organizing center polarization at the cytotoxic T lymphocyte/target cell synapse. Proc. Natl. Acad. Sci. U.S.A. 110, 6073–6078. 10.1073/pnas.1218640110 23536289 PMC3625254

[B7] Blanco B. Compte M. Lykkemark S. Sanz L. Alvarez-Vallina L. (2019). T cell-redirecting strategies to 'STAb' tumors: beyond CARs and bispecific antibodies. Trends Immunol. 40, 243–257. 10.1016/j.it.2019.01.008 30827461

[B8] Bonello G. Blanchard N. Montoya M. C. Aguado E. Langlet C. He H. T.. (2004). Dynamic recruitment of the adaptor protein LAT: LAT exists in two distinct intracellular pools and controls its own recruitment. J. Cell Sci. 117, 1009–1016. 10.1242/jcs.00968 14996932

[B9] Brinkmann U. Kontermann R. E. (2017). The making of bispecific antibodies. MAbs. 9, 182–212. 10.1080/19420862.2016.1268307 28071970 PMC5297537

[B10] Carpenito C. Milone M. C. Hassan R. Simonet J. C. Lakhal M. Suhoski M. M.. (2009). Control of large, established tumor xenografts with genetically retargeted human T cells containing CD28 and CD137 domains. Proc. Natl. Acad. Sci. U.S.A. 106, 3360–3365. 10.1073/pnas.0813101106 19211796 PMC2651342

[B11] Cemerski S. Das J. Giurisato E. Markiewicz M. A. Allen P. M. Chakraborty A. K.. (2008). The balance between T cell receptor signaling and degradation at the center of the immunological synapse is determined by antigen quality. Immunity 29, 414–422. 10.1016/j.immuni.2008.06.014 18760640 PMC3962836

[B12] Das V. Nal B. Dujeancourt A. Thoulouze M. I. Galli T. Roux P.. (2004). activation-induced polarized recycling targets T cell antigen receptors to the immunological synapse; involvement of SNARE complexes. Immunity 20, 577–588. 10.1016/S1074-7613(04)00106-2 15142526

[B13] Davenport A. J. Cross R. S. Watson K. A. Liao Y. Shi W. Prince H. M.. (2018). Chimeric antigen receptor T cells form nonclassical and potent immune synapses driving rapid cytotoxicity. Proc. Natl. Acad. Sci. U.S.A. 115, E2068–E2076. 10.1073/pnas.1716266115 29440406 PMC5834689

[B14] Ehrlich L. I. Ebert P. J. Krummel M. F. Weiss A. Davis M. M. (2002). Dynamics of p56lck translocation to the T cell immunological synapse following agonist and antagonist stimulation. Immunity 17, 809–822. 10.1016/S1074-7613(02)00481-8 12479826

[B15] Eshhar Z. Waks T. Gross G. Schindler D. G. (1993). Specific activation and targeting of cytotoxic lymphocytes through chimeric single chains consisting of antibody-binding domains and the gamma or zeta subunits of the immunoglobulin and T-cell receptors. Proc. Natl. Acad. Sci. U.S.A. 90, 720–724. 10.1073/pnas.90.2.720 8421711 PMC45737

[B16] Finney H. M. Lawson A. D. Bebbington C. R. Weir A. N. (1998). Chimeric receptors providing both primary and costimulatory signaling in T cells from a single gene product. J. Immunol. 161, 2791–2797. 9743337

[B17] Freiberg B. A. Kupfer H. Maslanik W. Delli J. Kappler J. Zaller D. M.. (2002). Staging and resetting T cell activation in SMACs. Nat. Immunol. 3, 911–917. 10.1038/ni836 12244310

[B18] Harwood S. L. Alvarez-Cienfuegos A. Nuñez-Prado N. Compte M. Hernández-Pérez S. Merino N.. (2017). ATTACK, a novel bispecific T cell-recruiting antibody with trivalent EGFR binding and monovalent CD3 binding for cancer immunotherapy. Oncoimmunology 7:e1377874. 10.1080/2162402X.2017.1377874 29296540 PMC5739562

[B19] Hashimoto-Tane A. Yokosuka T. Sakata-Sogawa K. Sakuma M. Ishihara C. Tokunaga M.. (2011). Dynein-driven transport of T cell receptor microclusters regulates immune synapse formation and T cell activation. Immunity 34, 919–931. 10.1016/j.immuni.2011.05.012 21703543

[B20] Huse M. Lillemeier B. F. Kuhns M. S. Chen D. S. Davis M. M. (2006). T cells use two directionally distinct pathways for cytokine secretion. Nat. Immunol. 7, 247–255. 10.1038/ni1304 16444260

[B21] Li J. Stagg N. J. Johnston J. Harris M. J. Menzies S. A. DiCara D.. (2017). Membrane-proximal epitope facilitates efficient T cell synapse formation by anti-FcRH5/CD3 and is a requirement for myeloma cell killing. Cancer Cell. 31, 383–395. 10.1016/j.ccell.2017.02.001 28262555 PMC5357723

[B22] Martín-Cófreces N. B. Baixauli F. Sánchez-Madrid F. (2014). Immune synapse: conductor of orchestrated organelle movement. Trends Cell Biol. 24, 61–72. 10.1016/j.tcb.2013.09.005 24119664 PMC4347664

[B23] Martín-Cófreces N. B. Robles-Valero J. Cabrero J. R. Mittelbrunn M. Gordón-Alonso M. Sung C. H.. (2008). MTOC translocation modulates IS formation and controls sustained T cell signaling. J. Cell Biol. 182, 951–962. 10.1083/jcb.200801014 18779373 PMC2528574

[B24] Martín-Cófreces N. B. Vicente-Manzanares M. Sánchez-Madrid F. (2018). Adhesive interactions delineate the topography of the immune synapse. Front. Cell Dev. Biol. 6:149. 10.3389/fcell.2018.00149 30425987 PMC6218456

[B25] Maude S. L. Laetsch T. W. Buechner J. Rives S. Boyer M. Bittencourt H.. (2018). Tisagenlecleucel in children and young adults with B-cell lymphoblastic leukemia. N. Engl. J. Med. 378, 439–448. 10.1056/NEJMoa1709866 29385370 PMC5996391

[B26] Milone M. C. Fish J. D. Carpenito C. Carroll R. G. Binder G. K. Teachey D.. (2009). Chimeric receptors containing CD137 signal transduction domains mediate enhanced survival of T cells and increased antileukemic efficacy *in vivo*. Mol. Ther. 17, 1453–1464. 10.1038/mt.2009.83 19384291 PMC2805264

[B27] Monks C. R. Freiberg B. A. Kupfer H. Sciaky N. Kupfer A. (1998). Three-dimensional segregation of supramolecular activation clusters in T cells. Nature 395, 82–86. 10.1038/25764 9738502

[B28] Mukherjee M. Mace E. M. Carisey A. F. Ahmed N. Orange J. S. (2017). Quantitative imaging approaches to study the CAR immunological synapse. Mol. Ther. 25, 1757–1768. 10.1016/j.ymthe.2017.06.003 28663103 PMC5542801

[B29] Murugesan S. Hong J. Yi J. Li D. Beach J. R. Shao L.. (2016). Formin-generated actomyosin arcs propel T cell receptor microcluster movement at the immune synapse. J. Cell Biol. 215, 383–399. 10.1083/jcb.201603080 27799367 PMC5100289

[B30] Neelapu S. S. Locke F. L. Bartlett N. L. Lekakis L. J. Miklos D. B. Jacobson C. A.. (2017). Axicabtagene ciloleucel CAR T-cell therapy in refractory large B-cell lymphoma. N. Engl. J. Med. 377, 2531–2544. 10.1056/NEJMoa1707447 29226797 PMC5882485

[B31] Nuñez-Prado N. Compte M. Harwood S. Álvarez-Méndez A. Lykkemark S. Sanz L.. (2015). The coming of age of engineered multivalent antibodies. Drug Discov. Today 20, 588–594. 10.1016/j.drudis.2015.02.013 25757598

[B32] Offner S. Hofmeister R. Romaniuk A. Kufer P. Baeuerle P. A. (2006). Induction of regular cytolytic T cell synapses by bispecific single-chain antibody constructs on MHC class I-negative tumor cells. Mol. Immunol. 43, 763–771. 10.1016/j.molimm.2005.03.007 16360021

[B33] Przepiorka D. Ko C. W. Deisseroth A. Yancey C. L. Candau-Chacon R. Chiu H. J.. (2015). FDA approval: blinatumomab. Clin. Cancer Res. 21, 4035–4039. 10.1158/1078-0432.CCR-15-0612 26374073

[B34] Purbhoo M. A. Liu H. Oddos S. Owen D. M. Neil M. A. Pageon S. V.. (2010). Dynamics of subsynaptic vesicles and surface microclusters at the immunological synapse. Sci. Signal. 3:ra36. 10.1126/scisignal.2000645 20460647

[B35] Schubert D. A. Gordo S. Sabatino J. J. Jr Vardhana S. Gagnon E. Sethi D. K.. (2012). Self-reactive human CD4 T cell clones form unusual immunological synapses. J. Exp. Med. 209, 335–352. 10.1084/jem.20111485 22312112 PMC3280872

[B36] Soares H. Lasserre R. Alcover A. (2013). Orchestrating cytoskeleton and intracellular vesicle traffic to build functional immunological synapses. Immunol. Rev. 256, 118–132. 10.1111/imr.12110 24117817

[B37] Stinchcombe J. C. Majorovits E. Bossi G. Fuller S. Griffiths G. M. (2006). Centrosome polarization delivers secretory granules to the immunological synapse. Nature 443, 462–465. 10.1038/nature05071 17006514

[B38] Varma R. Campi G. Yokosuka T. Saito T. Dustin M. L. (2006). T cell receptor-proximal signals are sustained in peripheral microclusters and terminated in the central supramolecular activation cluster. Immunity 25, 117–127. 10.1016/j.immuni.2006.04.010 16860761 PMC1626533

[B39] Watanabe K. Kuramitsu S. Posey A. D. Jr. June C. H. (2018). Expanding the therapeutic window for CAR T cell therapy in solid tumors: the knowns and unknowns of CAR T cell biology. Front. Immunol. 9:2486. 10.3389/fimmu.2018.02486 30416506 PMC6212550

[B40] Xiong W. Chen Y. Kang X. Chen Z. Zheng P. Hsu Y. H.. (2018). Immunological synapse predicts effectiveness of chimeric antigen receptor cells. Mol. Ther. 26, 963–975. 10.1016/j.ymthe.2018.01.020 29503199 PMC6080133

[B41] Yi J. Wu X. S. Crites T. Hammer J. A. 3rd. (2012). Actin retrograde flow and actomyosin II arc contraction drive receptor cluster dynamics at the immunological synapse in Jurkat T cells. Mol. Biol. Cell 23, 834–852. 10.1091/mbc.e11-08-0731 22219382 PMC3290643

